# Specific sialylation of N-glycans and its novel regulatory mechanism

**DOI:** 10.1007/s10719-024-10157-8

**Published:** 2024-07-03

**Authors:** Jianguo Gu, Tomoya Isaji

**Affiliations:** Division of Regulatory Glycobiology, Institute of Molecular Biomembrane and Glycobiology, Tohoku Medical and Pharmaceutical University, 4-4-1 Komatsushima, Aoba-ku, Sendai, Miyagi, 981-8558 Japan

**Keywords:** Focal adhesion kinase (FAK), N-Glycan, Golgi phosphoprotein 3 (GOLPH3), Integrin, Phosphatidylinositol 4-kinase (PI4K), Sialyltransferase, Sialylation

## Abstract

Altered glycosylation is a common feature of cancer cells. Some subsets of glycans are found to be frequently enriched on the tumor cell surface and implicated in different tumor phenotypes. Among these, changes in sialylation have long been associated with metastatic cell behaviors such as invasion and enhanced cell survival. Sialylation typically exists in three prominent linkages: α2,3, α2,6, and α2,8, catalyzed by a group of sialyltransferases. The aberrant expression of all three linkages has been related to cancer progression. The increased α2,6 sialylation on N-glycans catalyzed by β-galactoside α2,6 sialyltransferase 1 (ST6Gal1) is frequently observed in many cancers. In contrast, functions of α2,3 sialylation on N-glycans catalyzed by at least three β-galactoside α2,3-sialyltransferases, ST3Gal3, ST3Gal4, and ST3Gal6 remain elusive due to a possibility of compensating for one another. In this minireview, we briefly describe functions of sialylation and recent findings that different α2,3 sialyltransferases specifically modify target proteins, as well as sialylation regulatory mechanisms vis a complex formation among integrin α3β1, Golgi phosphoprotein 3 (GOLPH3), phosphatidylinositol 4-kinase IIα (PI4KIIα), focal adhesion kinase (FAK) and sialyltransferase, which suggests a new concept for the regulation of glycosylation in cell biology.

## Functions of sialylation

It has been well-known that glycans on the cell surface undergo dramatic changes upon carcinogenesis, which may link altered glycosylation to tumor behavior. Many studies have demonstrated the roles of different tumor-associated glycans in various stages of human cancer progression [1–3]. Among these, altered sialylation on the cell surface is crucial, as sialic acids are a diverse group of negatively charged monosaccharides typically found as terminal components attached to glycoconjugates, including N-glycans, O-glycans, and glycosphingolipids [4, 5]. Sialic acids have been found not only in mammals but also in lower vertebrates and invertebrates, indicating their evolutionary significance [6, 7]. In mammalian cells, the most common sialic acids are N-acetylneuraminic acid (Neu5Ac) and N-glycolylneuraminic acid (Neu5Gc), although only Neu5Ac is present in human cells due to a specific mutation [8]. To date, sialic acids are known to be linked via an α2,3 or α2,6 bond to Gal/GalNAc or α2,8 bond to sialic acid in glycoproteins through a group of sialyltransferases [9]. Given the relatively strong electronegative charge of sialic acids and their location at the outmost reaches of the cell surface, it is not surprising that sialic acids modulate the conformation and stabilization of proteins, interactions with other molecules around the environment, as well as normal processes including transmembrane receptors-mediated cellular signaling, fertilization, cell growth, differentiation, and apoptosis [10, 11].

On the other hand, altered sialylation has been associated with the malignancy of carcinoma. Many cancers associated with sialylated structures, which include sialyl Thomsen-nouvelle antigen (sialyl Tn), sialyl Lewis antigen (sLe), α2,6 sialylated lactosamine, polysialic acid, and gangliosides [12–16]. These structures can enhance cancer cell survival and metastatic potential. Altered expression of these structures in cancer cells could result from multiple mechanisms. Loss of expression or excessive expression of certain sialyltransferases is frequently observed in cancers and proposed to contribute to cancer progression [14]. High sialylation can protect cancer cells from recognition and eradication by the immune system [17]. This high sialylation is also closely related to tumor invasion and metastasis, as it affects cell-cell and cell-matrix interactions [18, 19]. The roles of different sialyltransferases in tumor progression have been comprehensively described [20, 21].

The β-galactoside α2,6 sialyltransferase 1 (ST6Gal1), an enzyme catalyzing the α2,6 sialylation on N-glycans, has been well investigated because the altered expression of ST6Gal1 is observed in many kinds of cancer cells and tissues [22–24]. Many studies have shown that the expression of ST6Gal1 is increased in diverse carcinomas, which may highly correlate with tumor progression [9]. For example, ST6Gal1 regulates macrophage apoptosis by controlling the α2,6-sialylation of tumor necrosis factor receptor-1 [25]. ST6Gal1 is up-regulated in colon carcinoma, and its metastasis and poor prognosis are ascribed to sialylation of the Fas death receptor by ST6Gal1 that protects Fas-mediated apoptosis [26]. Moreover, α2,6 sialylation is greatly enhanced in cancer stem cells and induced pluripotent stem (iPS) cells [27, 28], as well as epithelial-mesenchymal transition (EMT), in which α2,6 sialylation up-regulates integrin β1-mediated cell migration [29]. It is also reported that the differentiation of human dendritic cells is accompanied by an increased expression of sialylated glycans, mainly through the up-regulation of ST6Gal1 and others [30]. Interestingly, the GlcNAc branching of N-glycans may also influence different sialylation. For example, several studies showed that a low degree of branching favors α2,6 sialylation but does not favor α2,3 sialylation [31, 32]. Suppression of GlcNAc branch formation by overexpression of N-acetylglucosaminyltransferase III (GnT-III), which catalyzes the addition of the bisecting GlcNAc to block GlcNAc branching [33], significantly inhibits the α2,3 sialylation, but not the α2,6 sialylation [34].

## Specificities of three α2,3-sialyltransferases for target proteins

The α2,3-sialylation of N-glycans is considered essential but complicated because the functions of the three β-galactoside α2,3-sialyltransferases, ST3Gal3, ST3Gal4, and ST3Gal6, might be compensating for one another. ST3Gal3, ST3Gal4, and ST3Gal6 show a similar enzymatic substrate specificity in catalyzing the transfer of sialic acid on the terminal Gal residue of the disaccharide Galβ1–3/4GlcNAc of glycoproteins [21], which makes it plausible that the α2,3-sialylation modification of glycoproteins represents a co-involvement and/or compensation. And these three enzymes participate mainly in the generation of NeuAcα2,3Galβ3/4GlcNAc, which are the precursors of sLe^a^ or sLe^x^ [35, 36], two crucial tumor-associated sialylated glycoconjugates [37–39].

The expression of ST3Gal3 has been associated with tumor progression, differentiation, and metastasis in extrahepatic cholangiocarcinoma [40] and secondary tumor recurrence in gastric cancer [41]. ST3Gal6 is known to promote cell adhesion and migration of multiple myeloma cells [42]. On the other hand, there has been no consensus on the clinical significance of ST3Gal4. Some studies show a decrease in ST3Gal4 mRNA expression in gastrointestinal and ovarian cancer [43, 44]. In contrast, others have demonstrated significant increases in cervical intraepithelial neoplasia, colorectal cancer, and pancreatic adenocarcinoma tissues [45–47]. Furthermore, most studies only focused on the effects of three kinds of sialylation linkages on glycoproteins (α2,3, α2,6, and α2,8) or the expression levels of certain sialyltransferases in tumors. For example, α2,3-sialylation of α2 integrin has been related to the metastatic bone behavior of prostate cancer cells [48]; malignant transformation of the oral epithelium was found to be accompanied by α2,3-sialylation, wherein α2,6-sialylation was related to disease progression and metastatic potentials [49]; several sialyltransferase genes were found to be highly expressed in human colon, and gastric tumor tissues [50, 51]; and, essential functions of a single sialyltransferase have been observed by manipulation of the gene expression in a cell line [47, 52, 53]. However, very little is known about which sialyltransferase(s) is/are specifically responsible for synthesizing these altered glycans on defined glycoproteins and whether some sialyltransferases modulate favored glycoproteins to regulate their biological functions.

**Fig. 1:**
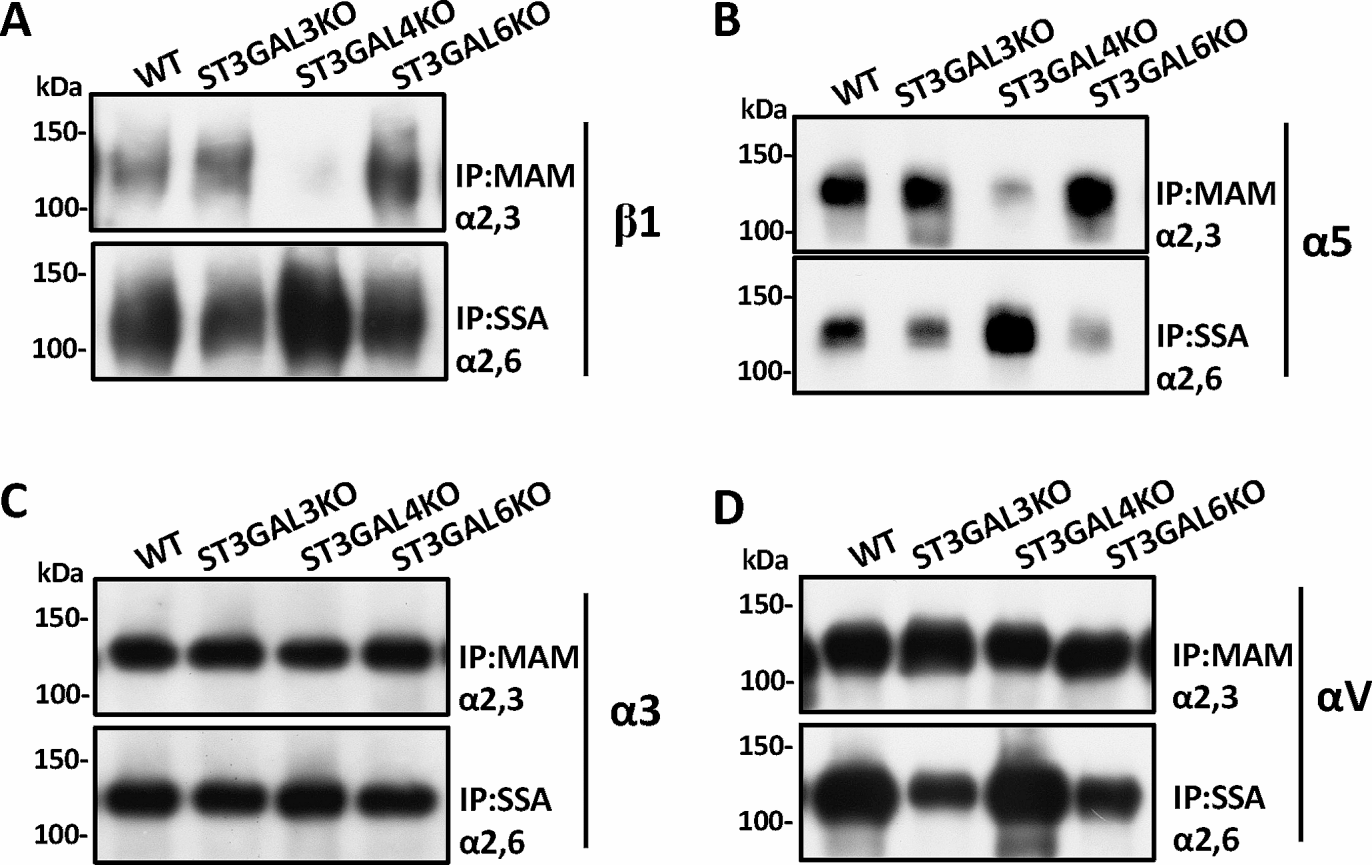
Comparison of sialylation levels on different integrin subunits among the three sialyltransferases’ knockout cells. Knockout (KO) of ST3Gal3, ST3Gal4, or ST3Gal6 gene in HeLa cells differed in altering the levels of ɑ2,3-sialylation and ɑ2,6-sialylation of specific glycoproteins. Equal amounts of cell lysates were immunoprecipitated with lectin MAM-agarose, which recognizes explicitly sialic acid ɑ2,3-sialylation, or lectin SSA-agarose, which specifically recognizes ɑ2,6-sialylation. Then, the immunoprecipitates were subjected to SDS-PAGE. The precipitated glycoproteins were immunoblotted for β1(**A**), ɑ5 (**B**), ɑ3 (**C**), and ɑv (**D**) integrin subunit antibodies

Recently, the respective functions of ST3Gal3, ST3Gal4, and ST3Gal6, and the three enzymes differed in their modification of the α2,3-sialylation of target proteins were described [54]. Qi, F. et al. used the CRISPR/Cas-9 system to establish individual gene knockout (KO) HeLa cell lines and restored the KO cells with the same or different genes to compare their functions. It clearly showed that ST3Gal4 specifically modifies the integrin β1 subunit, while ST3Gal6 modifies EGFR [54]. Consistently, the different modification was observed even in different integrin subunits (Fig. 1). The three α2,3-sialyltransferases showed negligible levels of compensation for ɑ2,3-sialylation on β1 or α5, but not α3 and αv subunits. Furthermore, ST3Gal4 could compete with ST6Gal1 for sialylation of the same target proteins, such as β1 and α5 subunits (Fig. 1). These observations may help understand their functional expressions and explain some previous results or interpret some controversial observations obtained from different cells or tumor tissues. In addition, the levels of p-AKT were only suppressed in the ST3Gal3 KO cells, suggesting that ST3Gal3 might specifically modify some glycoproteins that promote AKT activation to regulate cell proliferation [54]. Thus, the ranges for the α2,3-sialylation of glycoproteins affected by ST3Gal3, ST3Gal4, and ST3Gal6 differ. It has been reported that the sialylation of erythropoietin is modified explicitly by ST3Gal4 and not by either ST3Gal3 or ST3Gal6 in CHO cells [55].

Changes in the α2,6-sialylation levels of these KO cells merit recognition since α2,3- and α2,6-sialyltransferases may compete with common substrates to a certain extent. This competition is manifested in specific glycoproteins such as β1 and α5 integrin (Fig. 1). ST3Gal4-KO alone reciprocally increased the levels of α2,6-sialylation while the overexpression of ST3Gal4 conversely decreased the α2,6-sialylation levels of β1 integrin [54]. Therefore, evaluating the effect of α2,3-sialylation needs to consider the influences of α2,6-sialylation since the α2,6-sialylation of β1 plays essential roles in cell adhesion and cell proliferation [56, 57]. Therefore, understanding each substrate specificity of ST3Gal3, ST3Gal4, and ST3Gal6 is crucial to elucidate the biological functions of α2,3-sialylation and remains further studied.

## Effects of golgi phosphoprotein 3 (GOLPH3) on sialylation

The sialylation levels of glycoproteins on the cell surface have been mainly determined by the expression of sialyltransferases, sialidases, and substrate expression [58, 59]. Some transcription factors are critical for the transcriptional activation of the sialyltransferases in cancer cells. For example, the expression level of ST6Gal1 is up-regulated by Ras oncogene and thereby increases the α2,6 sialylation of β1 integrins, which promotes integrin-mediated cell adhesion, migration and proliferation [60, 61]. Thus far, sialylation levels mainly depend on their gene expression levels, while other mechanisms for regulation are usually neglected. However, it has been reported independently that GOLPH3, which has been identified as an oncogenic protein and increased in several human solid tumors [62], could anchor sialyltransferases to regulate sialylation on cell surface receptors without regulating gene expression levels of the sialyltransferases [63, 64]. In particular, the suppression of GOLPH3 attenuated the levels of cellular sialylation and integrin-dependent cell migration [64]. GOLPH3 triggered the incorporation of both core 2 *N*-acetylglucosaminyltransferase 1(C2GnT) [65] and ST6Gal1 into coatomer-coated (COPI) vesicles [63, 64]. Depletion of GOLPH3 led to an altered subcellular localization of these enzymes. In contrast, galactosyltransferase, an enzyme that does not interact with GOLPH3, was not incorporated into COPI vesicles [63, 64]. Recently, it was also reported that GOLPH3 and its paralogue GOLPH3L could bind the cytoplasmic tails of many glycosyltransferases through membrane-proximal positively charged residues by using binding studies, bioinformatics, and a Golgi retention assay [66]. Furthermore, the deletion of GOLPH3 and GOLPH3L caused multiple defects in glycosylation. Thus, GOLPH3 and GOLPH3L may be primary COPI adaptors that influence most of the glycosylation pathways of the Golgi.

GOLPH3 also has multiple cellular functions in vesicle trafficking and in support of Golgi apparatus structure, which has a specific affinity for phosphatidylinositol 4 phosphate (PI4P) catalyzed by phosphatidylinositol 4-kinase (PI4K) [67, 68]. Mammalian PI4Ks are classified as types II and III [69]. PI4KIIα is localized to the trans-Golgi network (TGN) [70]. Given the importance of GOLPH3 on sialylation and cell functions, it is plausible that PI4P expression at the TGN may influence sialylation.

## Regulatory mechanisms of sialylation by the integrin α3β1-GOLPH3-PI4KIIα- sialyltransferase axis

Integrins are heterodimeric cell surface adhesion receptors and major carriers of sialylation. The interaction between integrin and the extracellular matrix (ECM) is essential for cell adhesion, migration, viability, and proliferation [71, 72]. N-glycosylation is a critical regulator of integrin functions. For example, integrin α5β1 binding to fibronectin and integrin-mediated cell spreading and migration are modulated by the overexpression of glycosyltransferase genes such as GnT-III, -V, and ST6Gal1 [34, 73, 74]. In addition, sialylated N-glycans on the membrane-proximal domain of integrin β1 play crucial roles in integrin activation and in the complex formation between integrin and EGF receptors and syndecan-4 to regulate cell migration and proliferation [75].

**Fig. 2:**
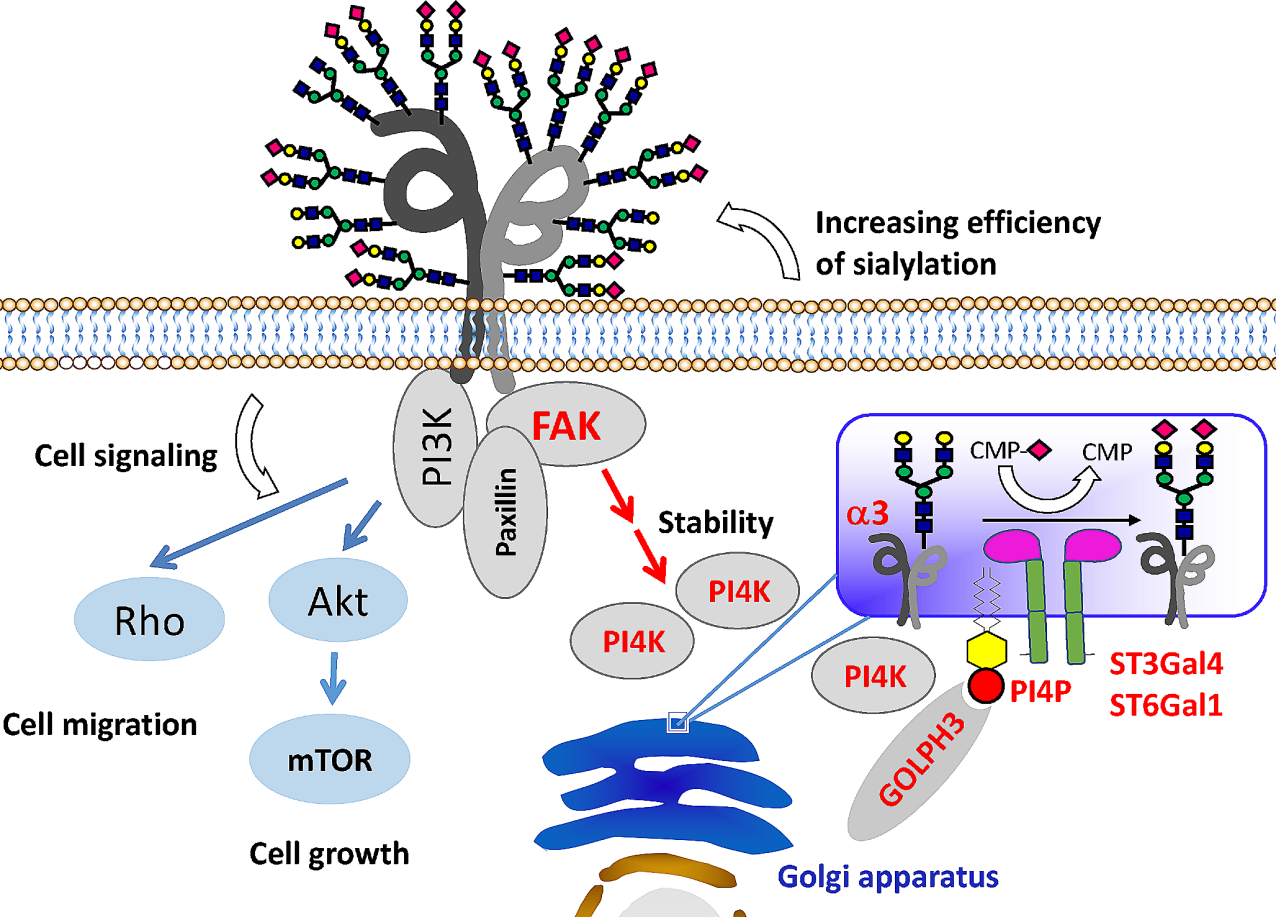
Schematic diagram of the proposed molecular mechanism for regulation of sialylation via the GOLPH3-PI4P-α3-sialyltransferases complex. Based on the effects of GOLPH3, PI4KIIα, integrin α3, and FAK on sialylation, and the complex formation among PI4KIIα, α3, and FAK or GOLPH3 and sialyltransferases enhances sialylation as previously described [64, 85, 86], we can propose that the sialylation is regulated by the integrin α3β1-FAK-GOLPH3-PI4P-sialyltransferase axis, which may provide a new concept for the regulation of glycosylation. Of course, further studies, such as the effects of integrin and/or FAK on sialylation, localization of sialyltransferases, and identification of their specific target glycoproteins, are required to clarify the underlying mechanism

In contrast to the regulation of integrin functions from the extracellular domain, it is well known that integrin function is regulated by its association with cytoplasmic molecules such as focal adhesion kinase (FAK) and phosphatidylinositol 3-kinase (PI3K) [76, 77]. The PI4K activity was detected in the immune complex with integrin β1, suggesting that integrin may also regulate the biosynthesis of PI4P [78–80]. PI4KIIα plays essential roles in clathrin-dependent molecular sorting and associates with TGN membranes [81–83], while PI4KIIIβ is enriched in the cis-medial Golgi in breast cancer cells [84]. To further understand the underlying mechanism for GOLPH3 expression on sialylation and cell functions, the effects of PI4KIIα have been investigated. Of particular interest, the over-expressions of either PI4KIIα or integrin α3, but not α5, greatly increased sialylation. Conversely, integrin α3-KO significantly inhibited sialylation in membrane proteins [85]. Both integrin α3β1 and PI4KIIα co-localized to the TGN where they physically interacted with each other, and PI4KIIα was specifically associated with integrin α3, but not α5. These results suggest a sialylation regulated by the axis, integrin α3β1-GOLPH3-PI4P-sialyltransferase (Fig. 2).

**Fig. 3:**
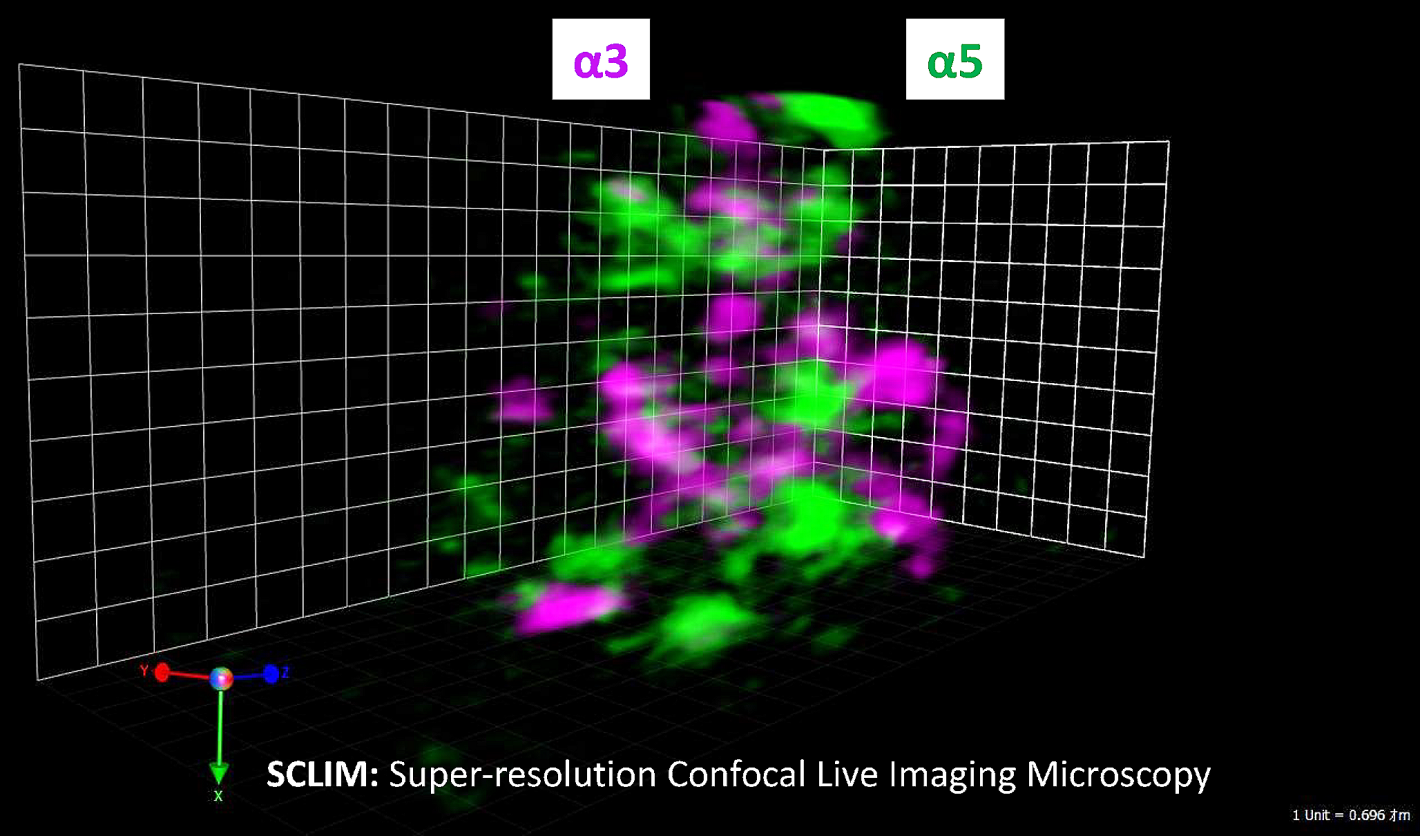
Spatial distributions of α3 and α5 at a cisterna of Golgi apparatus. HeLa cells expressing α5-GFP (green) with α3-mcherry (magenta) were observed with SCLIM. These 2D confocal fluorescence data were simultaneously collected for the two spectral window regions defined by band-pass filters (green, 490–545 nm; red, 580–660 nm). Volocity software (Perkin Elmer, MA) was used to reconstruct and deconvolve 3D images via point-spread functions optimized for SCLIM [87, 88]

These facts raise the question of why the interaction of PI4KIIα with integrin α3β1, but not that of α5β1, regulates the sialylation of N-glycans. Curiously, the localization of integrin α3 and α5 at cisterna of the Golgi apparatus was investigated by using an assay for dual cargo sorting into Golgi apparatus imaged by super-resolution confocal live imaging microscopy (SCLIM) [87, 88]. It clearly showed that integrin α3 and α5 were differently localized at different areas in the Golgi apparatus (Fig. 3), which may also explain the phenomena as observed in Fig. 1, in which α3 and α5 subunits were differently modified by α2,3- and α2,6-sialylation. Thus far, the association underlying the mechanism between α3β1 and PI4KIIα remains unclear. However, the specificity of α3β1 could be due to its interaction with the tetraspanin family, such as CD151, CD63, and CD9. It has been reported that integrin α3β1 and the tetraspanin family could interact with PI4KIIα [78–80]. Recently, we also found that FAK significantly increased PI4KIIα stability, which upregulated PI4P formation and contributed to the complex formation between GOLPH3 and sialyltransferase, finally promoting sialylation [86]. In addition, palmitoylation could also be a plausible factor since PI4Ks are proteins with membrane association and activity that is highly dependent on such a modification [89, 90].

Interestingly, the mass spectrometry analysis clearly showed that the complex between PI4KIIα and integrin α3 regulates the biosynthesis of sialylation on N-glycans, not O-glycans [85]. Although the molecular mechanism for the specificity remains unclear, it could not be excluded entirely from other possibilities for regulating *N*-glycan structures other than sialylation or gangliosides. Considering FAK is the downstream of integrin α3β1, but also α5β1, and different localization of α3 and α5 subunit at cisterna of Golgi apparatus (Fig. 3), questions such as whether integrins regulate sialylation on O-glycans or gangliosides may be answered after clarifying effects of FAK on sialylation.

## Conclusions and future directions

Given increasing evidence implicating sialylation in multiple pathological processes, including cancers, much effort has been undertaken during the last decade to clarify the functions of sialylation in cancer progression. Although a novel regulatory mechanism for sialylation is addressed in this review, defining regulatory mechanisms in detail and specific substrates for sialyltransferases will be necessary for a complete understanding of the roles of sialylation during cancer progression, which may provide new insights for targeting aggressiveness and drug resistance of cancer cells.

## Data Availability

They are provided as required.
